# A rare case of heterotaxy and left ventricular non-compaction in an adult

**DOI:** 10.5830/CVJA-2015-063

**Published:** 2016

**Authors:** A Chacko, L Scholtz, S Vedajallam, C van Wyk

**Affiliations:** Department of Radiology, Steve Biko Academic Hospital, University of Pretoria, Pretoria, South Africa; Scholtz & Partners, Diagnostic Radiologists, Pretoria, South Africa; Frere Hospital, East London, South Africa; Cardiologist in Private Practice, Zuid-Afrikaans Hospital, Pretoria, South Africa

**Keywords:** heterotaxy, dextrocardia, left ventricular non-compaction, LVNC, polysplenia, situs ambiguous, left isomerism

## Abstract

Heterotaxy syndrome with left ventricular non-compaction is a rare co-existence of abnormalities with unknown cause. It can be isolated with no other associations, or associated with congenital heart diseases, or it can occur with multiple other congenital abnormalities. We describe the third reported case of heterotaxy syndrome with left ventricular non-compaction presenting in an adult.

## Abstract

Heterotaxy, also known as situs ambiguous, is defined as the abnormal and disorganised arrangement of organs and vessels within the abdominal cavity. This is in contrast to the orderly arrangement that occurs in situs inversus or situs solitus.

The two major subcategories of situs ambiguous are situs ambiguous with polysplenia, and situs ambiguous with asplenia. Situs ambiguous with polysplenia, (which is also known as left isomerism or bilateral left-sidedness) is generally characterised by a midline position of the abdominal organs and multiple spleens/splenules.

Left ventricular non-compaction is a rare congenital abnormality of the heart with unknown cause. It can be isolated with no other associations, or associated with congenital heart diseases, or it can occur in conjunction with multiple other congenital abnormalities. The entity characteristically exhibits prominent and excessive trabeculae in the left ventricular wall segment, with the deep inter-trabecular recesses being perfused from the cavity.

Genetic causes associated with multiple gene mutations have been implicated in causing the arrest of normal embryogenesis within the endocardium and myocardium.^1^ Common clinical presentations include cardiac failure and tachyarrhythmia, as well as thromboembolic events. Associations with other cardiac and extra-cardiac abnormalities have been described.

We describe the third reported case of dextrocardia with left ventricular non-compaction, situs ambiguous with an interrupted inferior vena cava, and polysplenia presenting in an adult.

## Case report

A 47-year-old male patient presented to the cardiologist with a history of chronic atrial fibrillation and known dextrocardia on chest radiography. The main presenting symptom was dyspnoea on exertion. The patient was a smoker and had a history of high alcohol intake. On examination he was noted to be normotensive and with a normal resting heart rate with atrial fibrillation. Lung function tests showed a mild obstructive airways disease pattern.

Echocardiography confirmed the dextrocardia with hypertrophy, and possibly increased trabeculations were noted in the left ventricular wall. The ejection fraction was 50%, with a mildly enlarged left atrium and a normal-calibre left ventricular cavity.

On abdominal ultrasound, the liver was observed to be midline with extension into the left hypochondrium, and the patient was noted to have polysplenia with multiple splenules located in the right hypochondrium. The ultrasound also confirmed an absent inferior vena cava.

The patient’s blood work showed no abnormalities, with normal liver and renal function profile. Electrocardiography performed with a stress component showed no ischaemia and confirmed atrial fibrillation with no heart block.

**Fig. 1.**
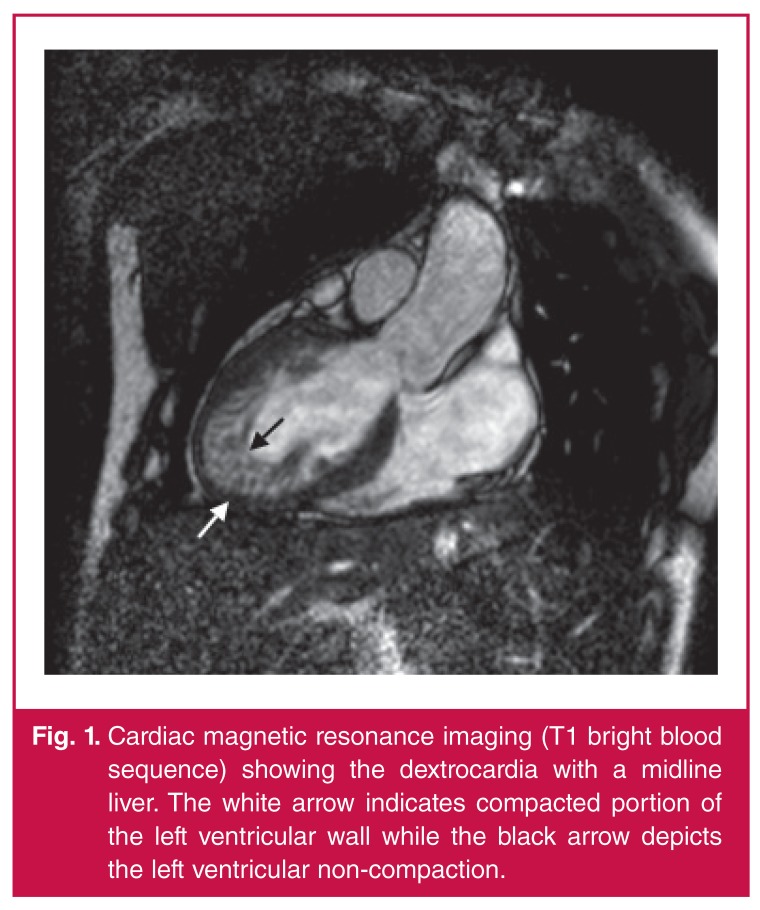
Cardiac magnetic resonance imaging (T1 bright blood sequence) showing the dextrocardia with a midline liver. The white arrow indicates compacted portion of the left ventricular wall while the black arrow depicts the left ventricular non-compaction.

**Fig. 2.**
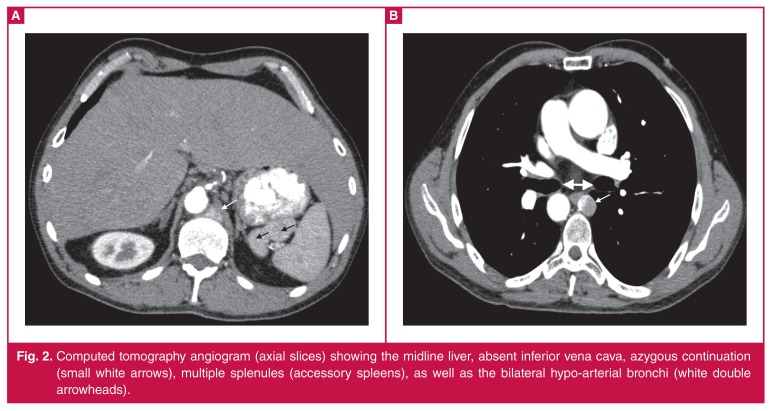
Computed tomography angiogram (axial slices) showing the midline liver, absent inferior vena cava, azygous continuation (small white arrows), multiple splenules (accessory spleens), as well as the bilateral hypo-arterial bronchi (white double arrowheads).

**Fig. 3.**
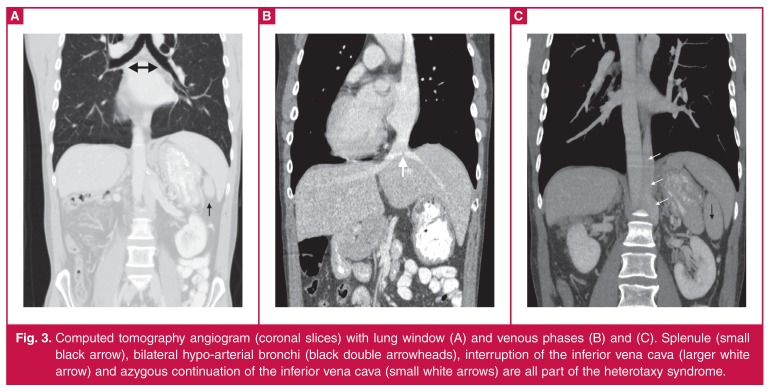
Computed tomography angiogram (coronal slices) with lung window (A) and venous phases (B) and (C). Splenule (small black arrow), bilateral hypo-arterial bronchi (black double arrowheads), interruption of the inferior vena cava (larger white arrow) and azygous continuation of the inferior vena cava (small white arrows) are all part of the heterotaxy syndrome.

Cardiac magnetic resonance (CMR) imaging was performed to further evaluate cardiac and great vessel structure and function (Fig. 1). Dextrocardia, heterotaxy, left isomerism and left ventricular non-compaction were confirmed on the CMR and subsequent computed tomography (CT) (Fig. 2, Fig. 3).

## Discussion

Dextrocardia is a cardiac positional anomaly in which the heart is located in the right hemithorax, with its base-to-apex axis directed to the right and caudally. The malposition is intrinsic to the heart and not caused by extra-cardiac abnormalities such as right lung hypoplasia, right pneumonectomy or diaphragmatic hernia.^2^

Heterotaxy, also known as situs ambiguous, is defined as the abnormal and disorganised arrangement of organs and vessels within the abdominal cavity. This is in contrast to the orderly arrangement that occurs in situs inversus or situs solitus. Patients with situs ambiguous and dextrocardia have associated congenital heart disease in 50 to 100% of cases, as opposed to patients with situs solitus or situs inversus and dextrocardia.^3^

The two major subcategories of situs ambiguous are situs ambiguous with polysplenia, and situs ambiguous with asplenia. Situs ambiguous with polysplenia (which is also known as left isomerism or bilateral left-sidedness) is generally characterised by a midline position of the abdominal organs and multiple spleens/splenules. Affected patients have a lower prevalence of congenital heart disease (50–90%) and less severe defects than those with situs ambiguous with asplenia.^4^

When evaluating a patient with dextrocardia on CT or MRI, a systematic and sequential approach has been suggested in order to fully evaluate abnormalities of the heart and vascular structures. The approach favoured by Maldjian and Saric^2^ is analysis of the following in sequence: viscero-atrial situs, atrioventricular connections, ventricular morphology, ventricular situs, chamber positions, ventriculo-arterial connections, and relationship of the great arteries. Finally, any associated anomalies, such as septal defects or pulmonic stenosis, should be described.

Situs of the viscera and atria is almost always concordant, and the atrial sinus is easily seen on cross-sectional imaging. On chest radiograph, this is also easily assessed by the location of the liver, spleen and stomach bubble. The morphology of the bronchial tree (usually best assessed on CT) is more accurate in determining atrial situs than the position of abdominal viscera. On chest radiographs in most patients, an enlarged azygous vein can be an indication of polysplenia, due to the high association with azygous or hemi-azygous continuation of the inferior vena cava.^4^

Evaluation of ventricular morphology, atrioventricular connections and relationships of the great arteries usually requires assessment by either CT angiography or MRI. The final step in analysis involves assessment of extra-cardiac abnormalities and possible syndromic associations. In patients who present as adults, the possible abnormalities are limited (other abnormalities generally presenting much earlier). Congenital heart and vascular defects that have been described include dextrocardia with situs inversus totalis (mirror image), situs solitus with normal relationship of great arteries (variation of dextroversion), situs solitus with levo- and/or dextrotransposition of the great arteries, and dextrocardia associated with polysplenia syndrome.^2^

Dextrocardia can also be associated with the heterotaxy syndromes of asplenia and polysplenia. Of the two syndromes, polysplenia is more likely to be associated with less severe cardiac malformations and therefore more likely to be encountered in adults. Up to 50% of cases of polysplenia syndrome can have dextrocardia. In polysplenia syndrome, there tends to be non-cyanotic congenital heart defects.^2^

Abnormalities associated with polysplenia syndrome are bilaterally symmetrical liver, bilateral bi-lobed lungs with bilateral hypo-arterial bronchi (left isomerism), bilateral superior vena cava, absence of the intrahepatic portion (interruption) of the inferior vena cava with azygous or hemi-azygous continuation, common atrium with complete absence of the atrial septum, endocardial cushion defect, hypoplasia or absence of one ventricle, valvular or subvalvular pulmonary stenosis, aortic stenosis or atresia, and double-outlet right ventricle. There is also an unexplained relationship between polysplenia and Kartagener’s syndrome.^2^

Left ventricular non-compaction (LVNC) is a hereditary primary cardiomyopathy with characteristic features of prominent trabeculations and conspicuous inter-trabecular recesses that penetrate deeply into the left ventricular myocardium, with a thin, compacted ventricular free wall (mainly in the affected areas), and diffuse systolic dysfunction with hypokinesia.^5^ The majority of reported cases describe involvement of the left ventricle, but the right ventricle and septum can also be affected.^6^

Non-compaction of the ventricular myocardium was first described in 1932, in an autopsy on a newborn.^7^ Since then, due to increasing awareness and continuously improving technology, the rates of diagnosis of LVNC have been steadily increasing. Imaging studies are the cornerstone of diagnosis of LVNC, with echocardiography being the main diagnostic tool. Computed tomography, angiography and magnetic resonance imaging (MRI) have been and can be used with equal success at diagnosis of the entity as well as for identification of associated abnormalities.

In the normally developed heart, the left ventricle has up to three prominent trabeculations and is less trabeculated than the right ventricle. In LVNC the trabeculations are more numerous (left ventricle compared to right ventricle) and thicker with deep recesses between the trabeculae.

Several diagnostic criteria have been proposed for LVNC, including a ratio of two for the wall thickness between the non-compacted trabeculated layer and the non-trabeculated compacted layer of the LVNC at end-systole, as measured along the parasternal short axis on echocardiography.^8^ Other criteria that can be used for diagnosis and possible classification include:^8^ (1) prominent and deep inter-trabecular recesses in the left ventricular lateral wall and apex, (2) direct blood flow from the ventricular cavity into the deep inter-trabecular recesses, as assessed by Doppler echocardiography, (3) two-layered structure of the ventricular wall, with an end-systolic ratio of non-compacted-to-compacted layer exceeding 1.4 (in infants), and (4) absence/presence of co-existing cardiac abnormalities.

The clinical presentation can vary and initially most children and adults are asymptomatic. The left ventricular function then gradually deteriorates and other presenting events may also occur, such as cardiac failure and thromboembolic events. The prognosis is poor, with patients facing the possibility of sudden death (due to cardiac arrhythmias, ischaemic strokes, etc.) or eventual death due to heart failure.^9^

The systolic dysfunction is thought to occur due to a relative ischaemia of the myocardium with a mismatch of myocardial oxygen supply and demand.^6^ Restricted myocardial perfusion and decreased coronary flow reserve, which suggests a coronary microcirculatory dysfunction, has been demonstrated previously by Jenni et al. with positron emission tomography (PET) 10

The multiple prominent trabeculations cause a restriction in filling, an abnormal ventricular relaxation pattern and diastolic dysfunction, with a generally poor eventual outcome for patients. Other complications can include thrombus formation within the recesses between trabeculae and subsequent thromboembolic events.

Delayed enhancement in the myocardium has been shown to increase under conditions of myocardial interstitial expansion or fibrosis.^11^ Previous histological studies have shown necrosis and fibrosis in patients with LVNC.^12^ These areas of fibrosis may serve as a focus or as foci for future lethal ventricular arrhythmias.

Cardiac MRI has proven very useful in identifying these areas of fibrosis for characterisation and further management (evaluation for heart transplant).^3^ No such foci were found in our patient (Fig. 1).

LVNC can be an isolated finding in the heart in the absence of other cardiac abnormalities. However, associations with other cardiac disorders, including coronary arterioventricular fistulae, ventricular septal defects, patent ductus arteriosus, atrial septal defects, a left coronary artery originating from the pulmonary artery, and dextrocardia have all been reported.^3^

Non-compaction of the myocardium can be either isolated or in conjunction with other congenital heart diseases. LVNC has been identified in relatively high association in patients with Ebstein’s anomaly with a reported figure of up to 18% of patients with Ebstein’s having non-compaction.^13^

Other associations previously reported include mitochondrial disorders, Barth syndrome, hypertrophic cardiomyopathy, muscular dystrophy type 1, 1p36 deletion, Turner syndrome, Ohtahara syndrome, distal 5q deletion, mosaic trisomy 22, trisomy 13, Di George syndrome, and 1q43 deletion with decreasing frequency, as well as Pierre-Robin syndrome.^6^ Malfunctioning of a rho-associated kinase has been implicated in the onset of the heterotaxy syndrome.^14^ Karyotyping and genetic testing have not been performed in our patient to date. CT angiography and/or MRI can be used in these patients to identify vasculature and other cardiac abnormalities, and associated congenital non-cardiac abnormalities.

## Conclusion

We report on only the third known case of dextrocardia, situs ambiguous with polysplenia, and left ventricular non-compaction in an adult. All the characteristic morphological features could easily be identified on imaging studies including but not limited to echocardiography, CT angiography and MRI.
